# Supplementary figures and images for: Clinical and Genetic Features of NR2E3-Associated Retinopathy: A Report of Eight Families with a Longitudinal Study and Literature Review
Source: Genes (Basel). 2023 Jul 26;14(8):1525. doi: 10.3390/genes14081525 (PMC10454055; doi:10.3390/genes14081525)

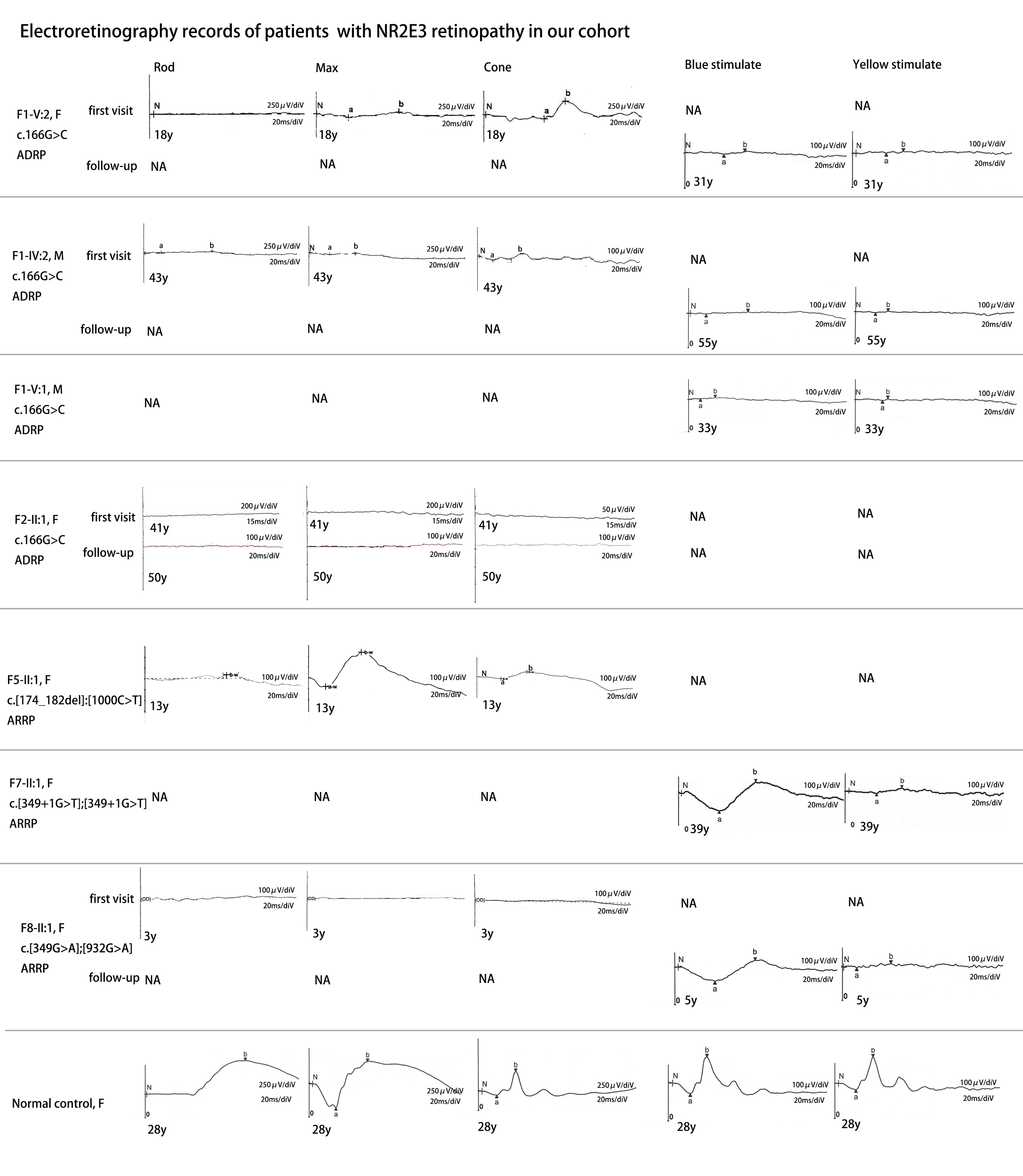

Supplement: Supplementary file 1 [file genes-14-01525-s001.zip › Supplementary figure S1-R1.tif]

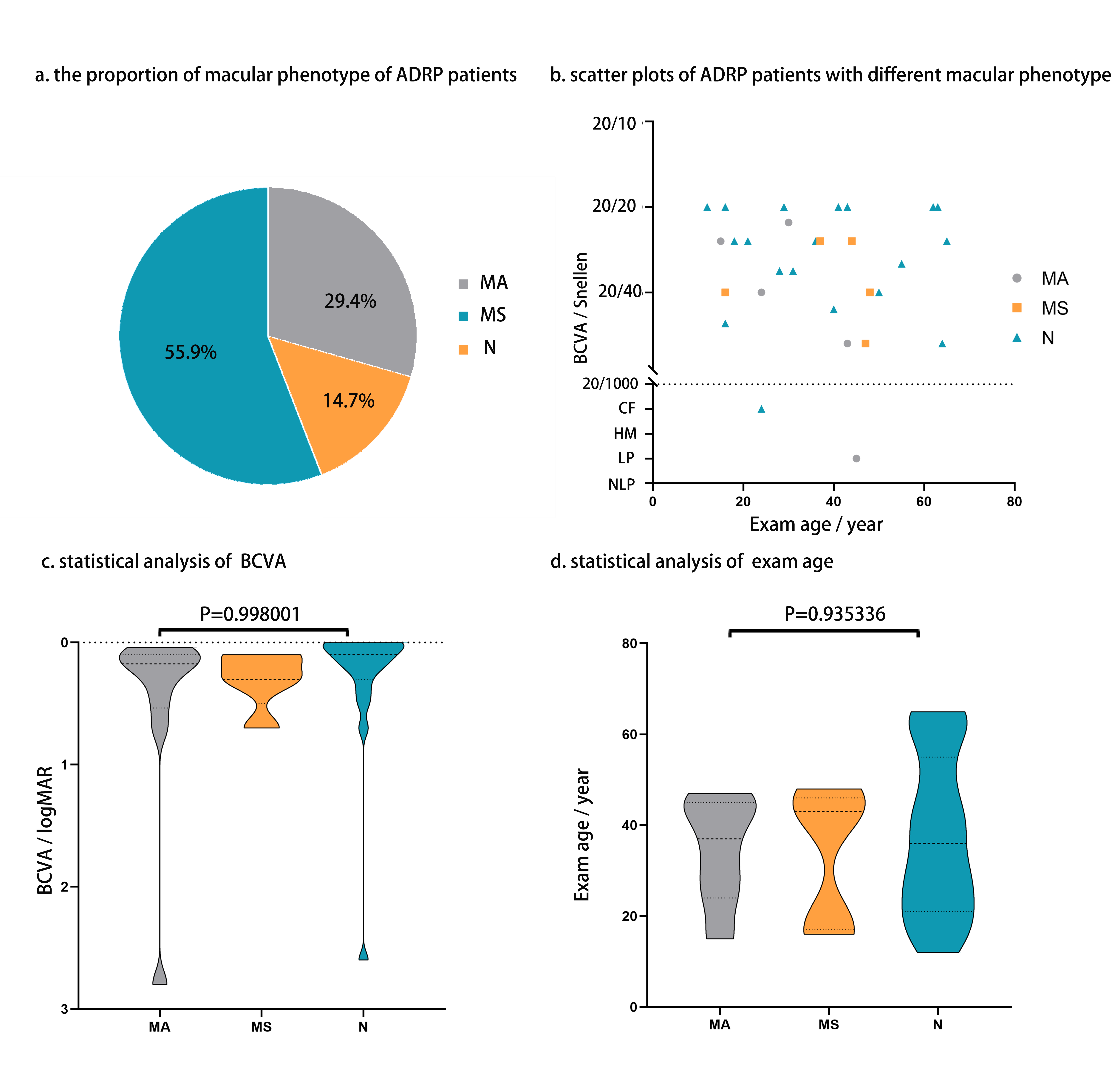

Supplement: Supplementary file 1 [file genes-14-01525-s001.zip › Supplementary figure S2-R1.tif]

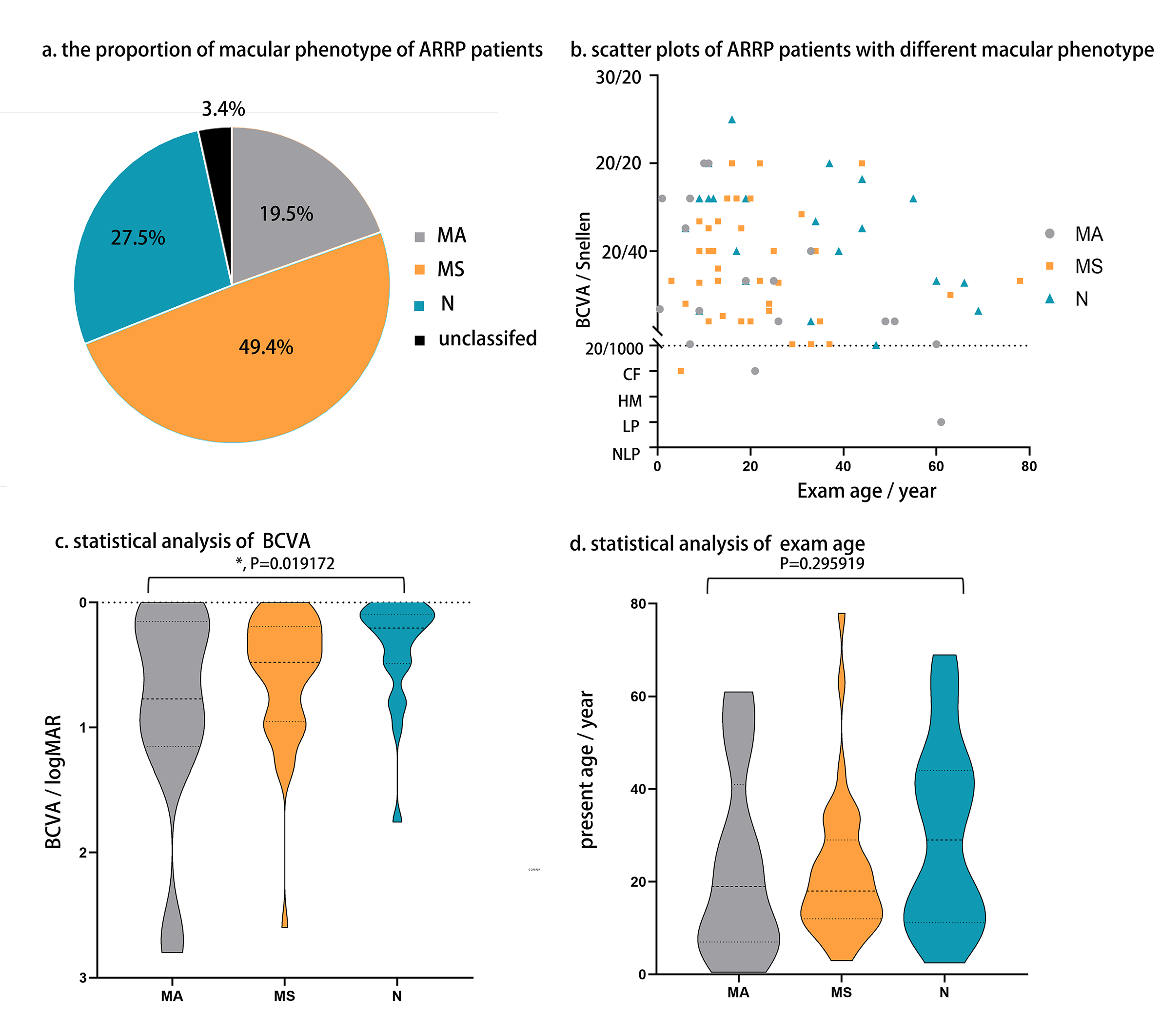

Supplement: Supplementary file 1 [file genes-14-01525-s001.zip › Supplementary figure S3-R1.tif]

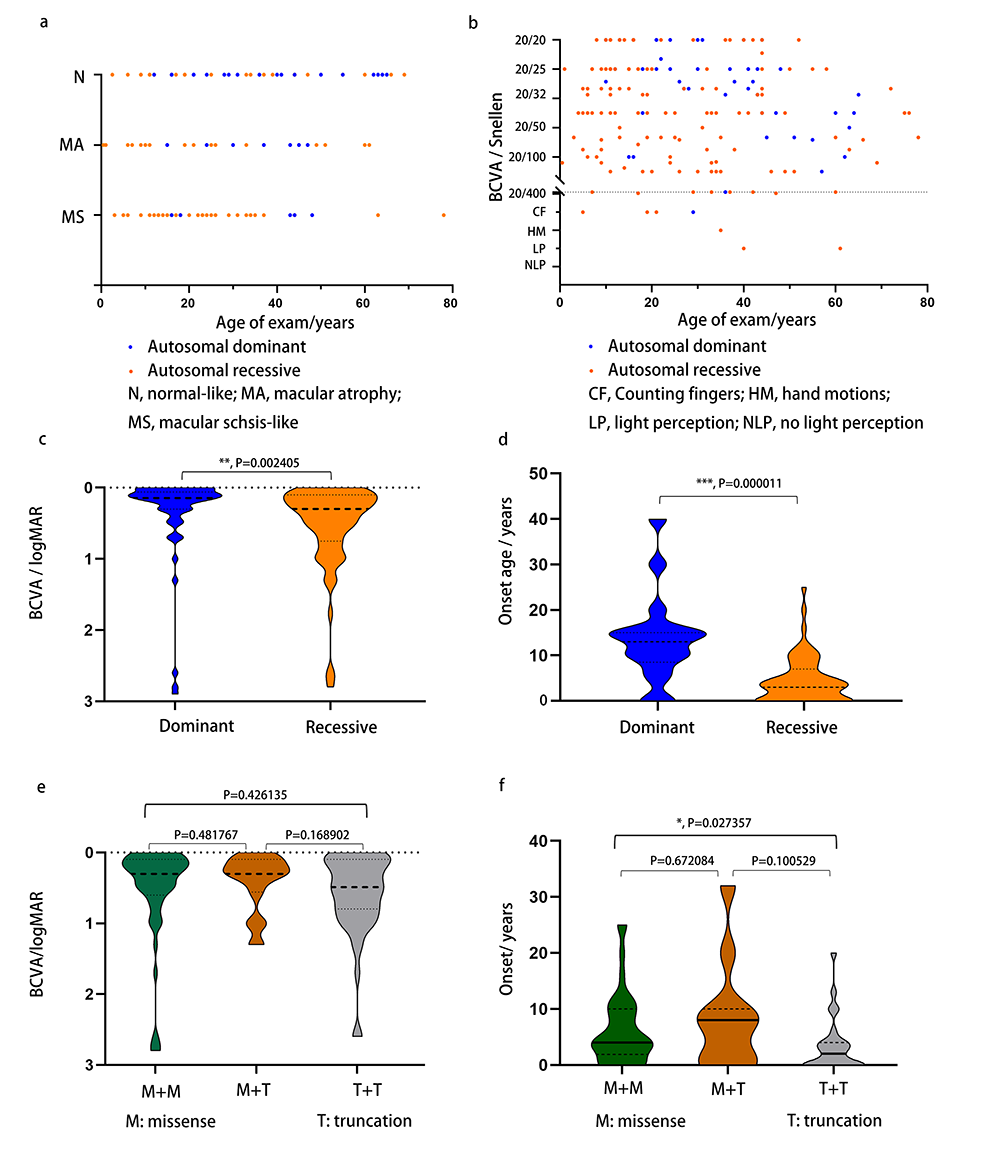

Supplement: Supplementary file 1 [file genes-14-01525-s001.zip › Supplementary figure S4-R1.tif]
